# Oxytocin-augmented group psychotherapy for negative symptoms in patients with schizophrenia spectrum disorders – a study protocol

**DOI:** 10.1192/j.eurpsy.2025.503

**Published:** 2025-08-26

**Authors:** M. Zierhut, N. Bergmann, I. Hahne, J. Wohlthan, M. Bajbouj, E. Hahn, K. Böge

**Affiliations:** 1 Psychiatry and Neurosciences, Charite - Universitätsmedizin, Berlin, Germany

## Abstract

**Introduction:**

New treatments for negative symptoms (NS) in schizophrenia spectrum disorders (SSD) are urgently needed. NS are believed to stem from disruptions between the mesocorticolimbic dopamine system and networks for socioemotional processes. Oxytocin (OXT) enhances connectivity between the neural networks, improving social cognition and NS. Lower plasma OXT levels are linked to greater NS severity and social cognition deficits in SSD. While OXT increases social cognition in healthy individuals, its effects in SSD are inconsistent. The social salience hypothesis suggests OXT’s effect varies with social context. Our pilot study showed reduced NS with OXT administration in a positive social setting using mindfulness-based group therapy (MBGT).

**Objectives:**

This trial aims to assess the effects of combining OXT with MBGT on each of the five NS in individuals with SSD. We hypothesize that OXT nasal spray administered before MBGT will significantly reduce NS compared to placebo. The primary outcome is the change in NS, measured by the Positive and Negative Syndrome Scale (T1 - T0 score difference) after 4 weeks and as secondary outcome on the Brief Negative Symptom Scale (BNSS) as well as changes of stress and affect.

**Methods:**

The research design is a triple-blinded, randomized, placebo-controlled study comparing OXT to placebo. Manual-based MBGT sessions, led by experienced psychotherapists, occur weekly for four weeks with groups of up to six patients. Participants receive intranasal OXT (24 I.U.) or placebo 30 minutes before sessions, aligning with the peak effect window (30-80 min) for optimal social behavior reinforcement. Plasma oxytocin levels are measured by radioimmunoassay. Recruitment will be at the Department of Psychiatry, Charité, Berlin, including both genders in mixed-sex groups, controlling for contraceptive use and menstrual cycle phase. Nasal sprays are indistinguishable. The primary outcome will be analyzed using ANCOVA, with treatment condition and training group as covariates. Based on pilot and previous study effects, a conservative effect size of f = 0.25 is assumed. With 1:1 randomization, 80% power, a 5% two-sided significance level, and a 10% drop-out rate, 140 subjects will be recruited.

**Results:**

This project explores how OXT augmentation enhances the positive effects of MBGT. It is expected that combining OXT with MBGT will significantly improve NS, stress, and affect in SSD patients. Preliminary results already show a significant reduction in social withdrawal and blunted affect in the OXT group compared to placebo.

**Image 1:**

**Figure:**
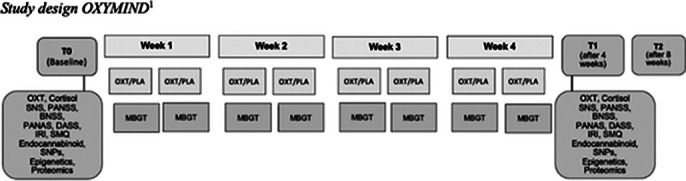

**Image 2:**

**Figure:**
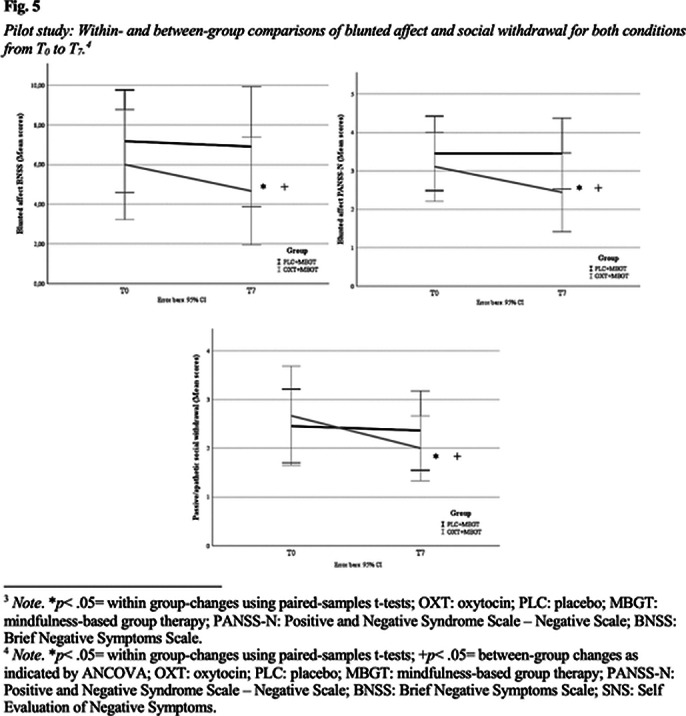

**Conclusions:**

Current treatments for NS in SSD are insufficient, highlighting the urgent need for new or combined strategies. Evidence supports the benefits of augmented psychotherapy. This project could pave the way for innovative, personalized psychiatric treatment for SSD.

**Disclosure of Interest:**

None Declared

